# Effect of prenatal stress and extremely low‐frequency electromagnetic fields on anxiety‐like behavior in female rats: With an emphasis on prefrontal cortex and hippocampus

**DOI:** 10.1002/brb3.2949

**Published:** 2023-03-21

**Authors:** Ehsan Hosseini, Davoud Kianifard

**Affiliations:** ^1^ Faculty of Veterinary Medicine Division of Physiology, Department of basic science Urmia University Urmia Iran; ^2^ Faculty of Veterinary Medicine Department of Basic Sciences University of Tabriz Tabriz Iran

**Keywords:** anxiety‐like behavior, BDNF, cas‐3, electromagnetic fields, female rat, GAP‐43, hippocampus, prefrontal cortex, prenatal stress

## Abstract

**Objective:**

Prenatal stress (PS) is a problematic situation resulting in psychological implications such as social anxiety. Ubiquitous extremely low‐frequency electromagnetic fields (ELF‐EMF) have been confirmed as a potential physiological stressor; however, useful neuroregenerative effect of these types of electromagnetic fields has also frequently been reported. The aim of the present study was to survey the interaction of PS and ELF‐EMF on anxiety‐like behavior.

**Method:**

A total of 24 female rats 40 days of age were distributed into four groups of 6 rats each: control, stress (their mothers were exposed to stress), EMF (their mothers underwent to ELF‐EMF), and EMF/stress (their mothers concurrently underwent to stress and ELF‐EMF). The rats were assayed using elevated plus‐maze and open field tests.

**Results:**

Expressions of the hippocampus GAP‐43, BDNF, and caspase‐3 (cas‐3) were detected by immunohistochemistry in Cornu Ammonis 1 (CA1) and dentate gyrus (DG) of the hippocampus and prefrontal cortex (PFC). Anxiety‐like behavior increased in all treatment groups. Rats in the EMF/stress group presented more serious anxiety‐like behavior. In all treatment groups, upregulated expression of cas‐3 was seen in PFC, DG, and CA1 and downregulated expression of BDNF and GAP‐43 was seen in PFC and DG and the CA1. Histomorphological study showed vast neurodegenerative changes in the hippocampus and PFC.

**Conclusion:**

The results showed ,female rats that underwent PS or/and EMF exhibited critical anxiety‐like behavior and this process may be attributed to neurodegeneration in PFC and DG of the hippocampus and possibly decreased synaptic plasticity so‐called areas.

## Graphical abstract

The present study showed that PS and ELF‐EMF could have potential hazardous implications in neurogenration in hippocamus and PFC in the pregnancy period and could result in anxiety‐like behavior in offspring. We also found for the first time that omnipresent ELF‐EMF not only induces neurodegeneration effects on hippocamus and PFC but also could exacerbate anxiey like behavior of PS, which may be attributed to hippocampal and PFC neurodegeneration and also neuroplasticity reduction.

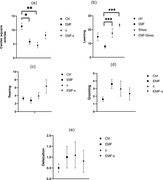

## 1 INTRODUCTION

Stressful life in the modern industrial world is an increasing problem and affects all of humanity, but especially pregnant women during the prenatal period (Dole et al., 2003; Gilles et al., 2018; van Heyningen et al., 2017). Exposure to stress during pregnancy can result in permanent behavioral changes and induces hypersensitivity to aversive stimuli as adult. Some studies have raised concerns that offspring of mothers exposed to stress during pregnancy may have an increased risk of specific diseases such as malformations, asthma, and mental and behavioral disorders (Cookson et al., 2009; Hansen et al., 2000; O'Connor et al., 2002). Prenatal stress (PS) can cause anxiety‐like behavior in rats (Miyagawa et al., 2011; Sun et al., 2013; Weinstock, 2017; Zuena et al., 2008) and has significantly harmful effects on development of fetus brain. Current studies on anxiety are typically in male animals, while female animals are often excluded because of interference of estrogen (Maghami et al., 2018; Smarr et al., 2017; Spritzer et al., 2013). In fact, there are reports that women are twice as likely as men to experience anxiety disorders and depression (Kuehner, 2017; Li & Graham, 2017), Because of this, we selected female rats to study changes in anxiety behavior as well as cellular and molecular changes in the hippocampus and prefrontal cortex. Studies demonstrate that the hippocampus and prefrontal cortex (PFC) are very sensitive to stress (McEwen et al., 2016; Suenaga et al., 2012). PS results in alteration of expression of the glucocorticoid receptors in the hippocampus which may be severely due to high‐stress hormone levels in fetus, produced by mother and reach to the fetus by crossing the placental barrier during stressful conditions (Mulder et al., 2002). Also, stress has been reported to decrease neurogenesis in hippocampus (McEwen et al., 2016), and also prenatally stressed offspring demonstrated a loss of neuron number in prefrontal cortex (Mychasiuk et al., 2012).

Caspase‐3 is a protein that has been found to be necessary for normal brain development as well as apoptosis that is initiated by hydrolyzing some cellular proteins resulting in protein degradation and eventually cell death (Creagh, 2014; Rybczynska et al., 2018) and also is involved in the development of synaptic plasticity (Gilman & Mattson, 2002) and the distinction of glial cells (Oomman et al., 2006). Additionally, PS leads to reduction in brain cell proliferation in the early days after birth with a simultaneous rise in cas‐3‐like activity in the hippocampus in offspring (Van den Hove et al., 2006). Also, it is established that Cas‐3 has a key role in initiating of apoptosis in prefrontal cortex neurons (Filipović et al., 2011).

One of the endogenous proteins of brain, which play a key role in proliferation and differentiation of new neurons and synapses, is brain‐derived neurotrophic factor (BDNF) (Huang & Reichardt, 2001). It is also known that BDNF plays a crucial role in modulation of both short‐ and long‐lasting synaptic interactions, namely synaptic plasticity (Biojone et al., 2015; Kowiański et al., 2018; Messaoudi et al., 2002). It is active in different areas of the brain including the hippocampus and the cortex (Yamada & Nabeshima, 2003). It has been revealed that BDNF is effective in the regulation of anxiety behavior. For example, BDNF knockout mice, which have low level of BDNF in the forebrain, showed high degrees of anxiety (Rios et al., 2001) and it has been demonstrated that overexpression of BDNF in hippocampus decreased anxiety‐like behaviors (Bahi, 2017). In one hand, PS results in a decrease in BDNF expression in the hippocampus of rat (Boersma et al., 2014; Weinstock, 2017) and on the other hand, it was shown that decreased hippocampal BDNF is involved in anxiety‐like behavior of rats (Saffarpour et al., 2019; Suliman et al., 2013). Also, the essential role of BDN in neuroplasticity has been proven (Begni et al., 2016; Lin & Huang, 2020) and BDNF is postulated as synaptic plasticity associated protein (Bramham & Messaoudi, 2005; De Vincenti et al., 2019; Leal et al., 2014). BDNF activates tyrosine kinase receptors and subsequently promotes neuronal survival, neuroplasticity, and synaptogenesis through different signaling pathways. Activated Trk receptors recruit tyrosine (Yang et al., 2020). Therefore, we assayed BDNF as a criterion for estimation of neuroplasticity.

The neuronal growth‐associated protein (GAP‐43) was primarily figured out to be related with nerve growth (Skene & Willard, 1981). It is a major protein kinase C substrate and is involved in neurite formation, regeneration, and plasticity (Aarts et al., 1998; Benowitz & Routtenberg, 1997). In addition, GAP‐43 has been found to be strictly associated with hippocampal (Zhu et al., 2019) and prefrontal cortex (Hrdina et al., 1998) synaptic plasticity. Mild stress has been shown to decrease GAP‐43 in the DG and the CA1 regions of the hippocampus of rats and subsequently reduce hippocampal plasticity (Huang et al., 2021). Besides, it has been suggested that prenatal stress results in upregulation of GAP‐43, which was considered as a protective mechanism against the toxicity of maternal stress hormone (Jutapakdeegul et al., 2010). We also measured GAP 43 as another criterion for evaluation of neuroplasticity along with BDNF.

Extremely low‐frequency (ELF 3−3000 Hz) (Garip & Akan, 2010) electromagnetic fields are generally existing in daily life all over the world and almost the majority of the human community are exposed to ELF‐EMF emanating by power lines, electrical panels, transformers, and domestic, electrical, and electronic devices (Grellier et al., 2014; Ivancsits et al., 2002; Karasek & Woldanska‐Okonska, 2004). Numerous studies have been performed on the behavior of animals which indicate that ELF‐EMF is safe in some cases (Burman et al., 2018; Lai et al., 2016b) but there are abundant studies declaring hazardous effects of them in promoting anxiety and depression behaviors in animals (Djordjevic et al., 2017; Hosseini, 2021; Karimi et al., 2019; Szemerszky et al., 2010) and humans (Hosseinabadi et al., 2020; Singh & Kapoor, 2014). Also, anti‐stress effect of ELF‐EMF has been reported (Nafisi et al., 2009). Besides, neuroregenerative effects of ELF‐EMF in hippocampus were reported by several studies (Cuccurazzu et al., 2010; Cuccurazzu et al., 2010; Sakhaie et al., 2017a; Tasset et al., 2012). Furthermore, ELF‐EMF has been found to increase neuroplasticity (Cichon et al., 2020; Cuccurazzu et al., 2010). In this respect, ELF‐EMF can act like a double‐edged sword. Of course, this duality may be due to differences in the properties of the electromagnetic fields used, such as power and frequency and also duration of exposure. We chose an electromagnetic field with a frequency of 60 Hz because this frequency is produced by most electrical home appliances (Mezei et al., 2001; Moriyama & Yoshitomi, 2005). In current study, we assayed BDNF, GAP‐43 and Cas‐3 of hippocampus and prefrontal cortex, which has been presented in this article. Since prenatal stress is one of the most common factors in inducing anxiety‐like behavior in offspring in modern societies (Said et al., 2015; Soares‐Cunha et al., 2018; Sofiabadi et al., 2021) and on the other hand due to the confinement of modern living environment with ELF‐EMF and the anxiogenic effect of ELF‐EMF (Djordjevic et al., 2017; Karimi et al., 2019; Miyagawa et al., 2011) and also considering the key role of the hippocampus and prefrontal cortex in anxiety‐like behavior, we hypothesized that if these two factors may have synergic effect of inducing anxiety‐like behavior via structural changes in hippocampus and prefrontal cortex or the interaction of these two factors can reduce their ability to create anxiety‐like behavior.

At present study for the first time, we studied the combined effect of PS and ELF‐EMF, two prevalent and pervasive environmental interferer, on anxiety‐like behavior in female offspring with immunehistochemical exploration of the hippocampus and PFC synaptic plasticity proteins and also histomorphological study of the hippocampus and PFC.

## 2 MATERIALS AND METHODS

### 2.1 Animals

Twenty‐four female Wistar rats of 3 months of age and weighing 200–250 g were brought from the Tabriz University Animal Care Center and were maintained for a week previous to the experiment at 25°C and 12 h light and 12 dark conditions for acclimation to the laboratory environment. Throughout this period, the rats were permitted to access to food and water ad libitum. All measures and tests were executed in accordance with the guidelines of the Tabriz Medical University Ethical Committee for protection of animals in research (IR.TBZMED.VCR.REC.1397.230) under the rules of the National Institutes of Health.

### 2.2 Experimental design

Female Wistar rats (as future mothers) were haphazardly allocated into four groups of 6 rats each (*n* = 6): future mothers of control group (C), future mothers of electromagnetic field (EMF) group, future mothers of stress group (S), and future mothers of EMF‐S groups (EMF/S). Dames in the control group (as future mothers of control groups) were exposed to a switched‐off jammer device. Female rats in the EMF group (as future mothers of ELF‐EMF group) were treated by ELF‐EMF (50 Hz, 100 μT) for 21 days, 4 h each day from 10:00 a.m. to 14:00 p.m. Female rats in the S group (as future mothers of stress group) were treated to various kind of stressors for 21 days (Table 1). The EMF‐S group female rats (as future mothers of EMF‐S group) were exposed to ELF‐EMF and stressors at the same time for 21 days. After this 21‐day phase, mating was allowed between male and female rats (each female rat with 2 male rats in a cage). First, the vaginal plaque was tested and after confirming the pregnancy of female rat, we put the pregnant rat to be exposed to second 21‐day phase of stress (group S) or electromagnetic field (group EMF) or both stress and electromagnetic field (group EMF‐S). All offspring were weaned on day 21 and then female descendants were chosen for behavioral tests and calculating the considered factors. Behavioral tests were executed at the age of 40 days and sampling was performed on day 41 (Flow chart 1). Therefore, generally we had 4 experimental groups, as follow: control (whose mothers located in switched out electromagnetic field device), stress (whose mothers underwent chronic stress), EMF (whose mothers were exposed to ELF‐EMF), and EMF/stress (whose mothers were simultaneously exposed to chronic stress and ELF‐EMF).

**FLOW CHART 1:**
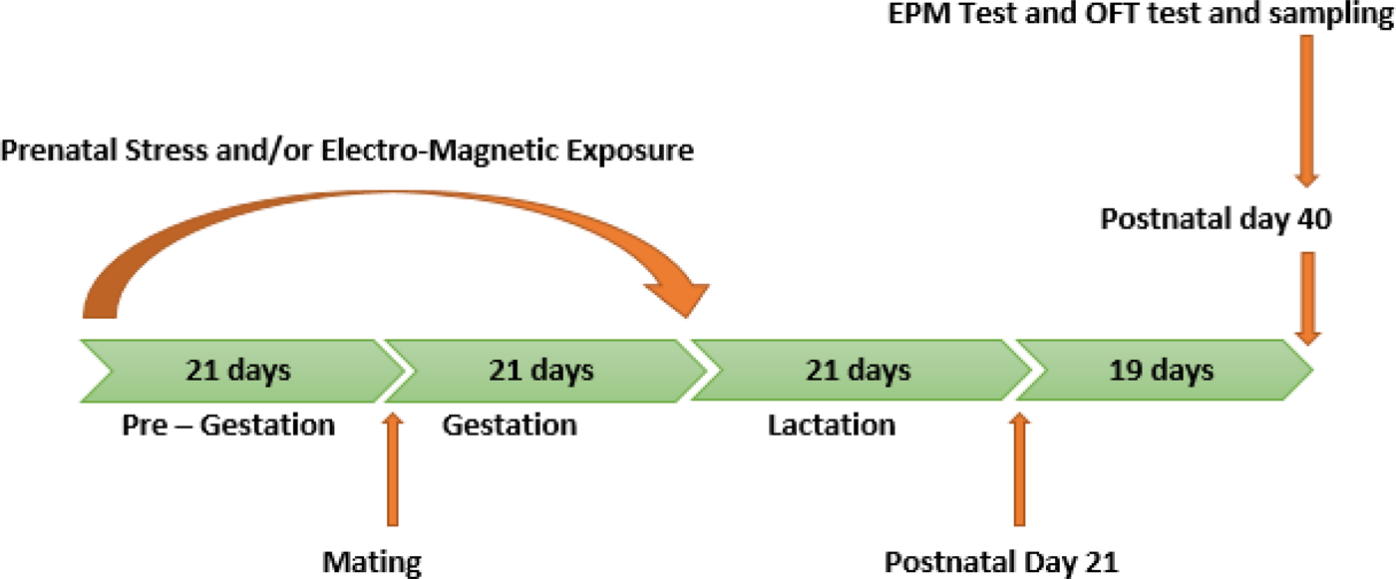
Timeline of the research, EPM: Elevated plus maze, OFT: Open field test.

**TABLE 1:** Stress protocol.

Days	Light/darkness	Multiple stress procedures timing
Sunday	Lights on overnight	Strobe light on 10.00 Room light off 10.00 Untilt cages 10.00 Strobe light off 16.00 Room lights on 16.00 Soil bedding 16.00 Start food and water deprivation 16.00
Saturday	Lights on overnight	Dry cage 11.00 Add water bottle 11.00 Food ad libitum 12.00 Remove water bottle 12.00 White noise on 12.00 White noise off 15.00
Monday	Lights off overnight	Empty water bottle 10.00 Add water bottle 11.00 Strobe lights on 17.00
Tuesday	Lights off overnight	Strobe lights off 10.30 Remove food 10.30 Tilt cages 10.30 White noise on 15.30 Paired housing 15.30 Untilt cages 15.30 White noise off 23.30
Wednesday	Lights off overnight	Rehouse singly 10.00 Add food ad libitum 12.00
Thursday	Lights on overnight	Start food and water deprivation 19.00
Friday	Lights on overnight	Restore food and water 14.30 Tilt cages 14.30

### 2.3 Stress protocol

The stress procedure was designed based on the technique of Lewitus (Lewitus et al., 2009) with some alteration. In the present study, the stress period was induced 3 weeks before and 3 weeks during pregnancy. Three weeks of stress is defined as chronic stress for rats in previous studies (Katz et al., 1981; Lewitus et al., 2009).

Each week the stress procedure consists of eight different stress modes (Table 1) (Hosseini et al., 2022): two periods of stroboscopic lighting (300 flash/min), one period of dry cage, two periods of noise (80 db), two periods of 45° cage tilt, one period of pair housing, and periods of water and food deprivation in which water deprivation was empty water bottles and food deprivation was followed by limited food intake (Hosseini et al., 2022; Salehpour et al., 2016).

### 2.4 Electromagnetic field device

**FIGURE 1:**
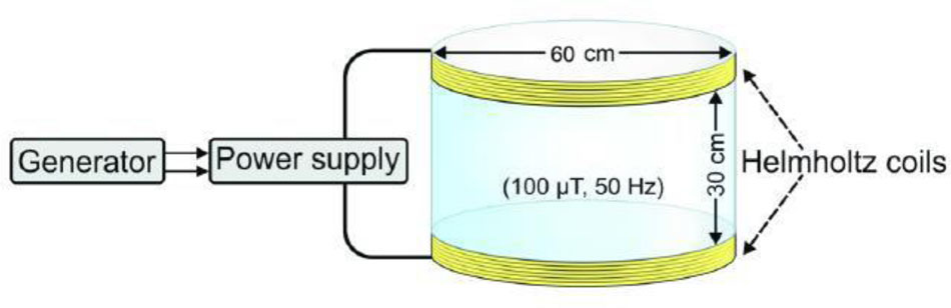
Helmholtz‐coil apparatus for the EMF exposure.

The magnetic field device used in our study is based on Helmholtz's screw theory designed in a physiology laboratory (Figure 1). The field‐generating part of this machine consists of two coils with a radius of 30 cm located coaxially at 30 cm distance from one another. The material of the rings containing coils is wooden and no metal was used in the mentioned distance, with a wooden tripod embedded on a sheet of foam. To produce a uniform field throughout this complex no metal parts were used. The magnetic field intensity was measured at the center of the space between two coils and also different parts of chamber by a digital Tesla Meter (Lutron 828, Taipei, Taiwan) before exposure. The field was homogeneous from a distance of 7 cm on each side towards the center of the chamber. The cages were located inside this zone. The strength of the ELF‐EMF was 100 μT in the homogenous zone. In this study the induced current was 50 Hz at an intensity of 100 μT (Hosseini et al., 2022; Podaru et al., 2015).

### 2.5 Elevated plus‐maze

Elevated Plus Maze device was used to evaluate anxiety. It is a standard model for evaluating the level of anxiety in rodents. This device is made of wood and includes two Open arm (each 50 × 10 cm) and two closed arms (each one 40 × 10 × 50 cm) and one central part (10 × 10 cm) with open arms facing each other. Each rat was separately transferred to the room half an hour before the test to be adapted to the room environment. Then for 5 min the rats were located in the central part so that they could move without restrictions in different parts of the maze. For doing the test, the following parameters were measured by observation: (1) the number of entries to the open arms; (2) the number of entries to the closed arms; (3) the total time spent in the open arms; (4) the total time spent in the closed arms; (5) the percentages of open arm entries (OAE %); and (6) the percentage of time spent in the open arms (open arm time, OAT %). These were calculated for each animal as follows: OAE% (the ratio of entries into open arms to total entries × 100); OAT% (the ratio of times spent in the open arms to total times spent in any arms × 100). The OAE% and OAT% are defined as anxiety indices (Sanders et al., 2003).

### 2.6 Open field tests (OFT)

For OFT a square apparatus 100 cm in length and width and also 30 cm in height was used. The internal area of the apparatus was divided into 25 equal square of 20 × 20 cm. In brief, after half hour adaption to the testing room, rats were put into the central area of the apparatus and monitored for 5 min (Crusio et al., 2013) as follows: (1) the number of standing of animals leaning on the wall (one or two paws in contact with the wall); (2) the number of rearing (standing on its two hind paws without touching the walls); (3) the number of grooming behaviors (face cleaning, paw licking, fur licking, head scraping, and rubbing); (4) the number of defecations; and (5) center square entries (frequency with which the rat enters the center region with all four paws). All these experiments were recorded with a camera connected to the recording and analysis system. The camera was put on the right top of the apparatus. The system and the experimental instruments were placed in a separated room to avoid of interfering factors.

### 2.7 Immunohistochemistry

The embedded brain tissues were cut into 5 μm sections. First, after dewaxing, the sections were immersed in 0.01 M sodium citrate buffer solution at 95−100°C for 15 min and then incubated with 3% hydrogen peroxide treatment for 10 min. Second, after being incubated with goat serum working solution for 15 min, the sections were incubated with anti‐rabbit BDNF antibody (EPR1292), anti‐rabbit cas‐3 antibody (D3R6Y) and anti‐rabbit GAP43 antibody (sc‐17790) at 4°C overnight. On the following day, the sections were incubated with biotinylated IgG, streptavidin, and DAB following the DAB detection kit (Abcam 64261, Cambridge, UK). The sections were digitized and analyzed by a Light microscope (Olympus AX70 Provis, Japan). Each section was imaged at 400× magnification and measured the percentage of positive area in each field of view by quantitative image analysis with Image J 1.45 software.

### 2.8 Histomorphometry

Formalin‐fixed midsagittal‐sectioned brain specimens were dehydrated using ascending grades of ethanol, cleared in xylene, and embedded in paraffin blocks. Then, serial sections of 5 μm thick were cut and stained with H&E (Suvarna et al., 2018). The prefrontal cortex and hippocampus were examined by a light microscope (Olympus AX70 Provis, Japan). The measurements was performed on the images obtained via AmScope digital camera (AmScope MD500).

### 2.9 Sampling

After the behavioral tests, all the animals were anesthetized with an intraperitoneal injection of ketamine hydrochloride and xylazine (60 and 12 mg/kg, respectively), sacrificed and the brains were isolated, then the hippocampus and prefrontal cortex were sequestered and frozen at −80°C. All sampling was performed at 10:00 a.m.

### 2.10 Statistically analysis

Normality of the data distribution were checked and confirmed using the Shapiro–Wilk test. As we want to check the simultaneous effect of two factors (ELF‐EMF and prenatal stress), on dependent variables (Anxiety‐like behavior and BDNF and others) separately (Assaad et al., 2015), we used two‐way analysis of variance (ANOVA). So, the data were analyzed using SPSS (version 25) via two‐way analysis of variance (ANOVA), followed by Tukey's post hoc test. *p* < .05 was considered significant. Furthermore, since we want to make pairwise comparisons between group means while the sample sizes for each group of our study are equal we performed Tukey's post hoc test.

## 3 RESULTS

### 3.1 Elevated plus‐maze test showed anxiety‐like behavior in all treatment groups

EMF and Stress group showed a decrease of OAT% (percentage of open arm time) in comparison with control (*p* < .001) and EMF/S group exhibit a decline of OAT% in compared to stress group (*p* < .05). OAE% (percentage of open arm entries) in EMF and stress group decreased compared to control (*p* < .001) and in EMF/S group decreased compared to stress group (*p* < .01) (Table 2).

**TABLE 2:** Effect of EMF on open arm time (%OAT) and open arm entries (%OAE) in plus‐maze test after prenatal stress.

	Control	ELF‐ EMF group	Stress group	EMF/Stress group
**Percentage of open arm time (%OAT)**	45.33 ± 0.95	26.33 ± 0.88***	27.83 ± 1.1***	21.50 ± 0.99 ^**#**^
**Open arm entries (%OAE)**	42.67 ± 1.02	30.67 ± 1.11***	32 ± 1.06***	24.5 ± 2.23^**##**^

ANOVA, followed by Tukey's post hoc test: ***p < .001 versus control group; ##*p* <.01; versus stress group. #*p* <.05; versus stress group. ANOVA, analysis of variance.

### 3.2 Open field test

**FIGURE 2:**
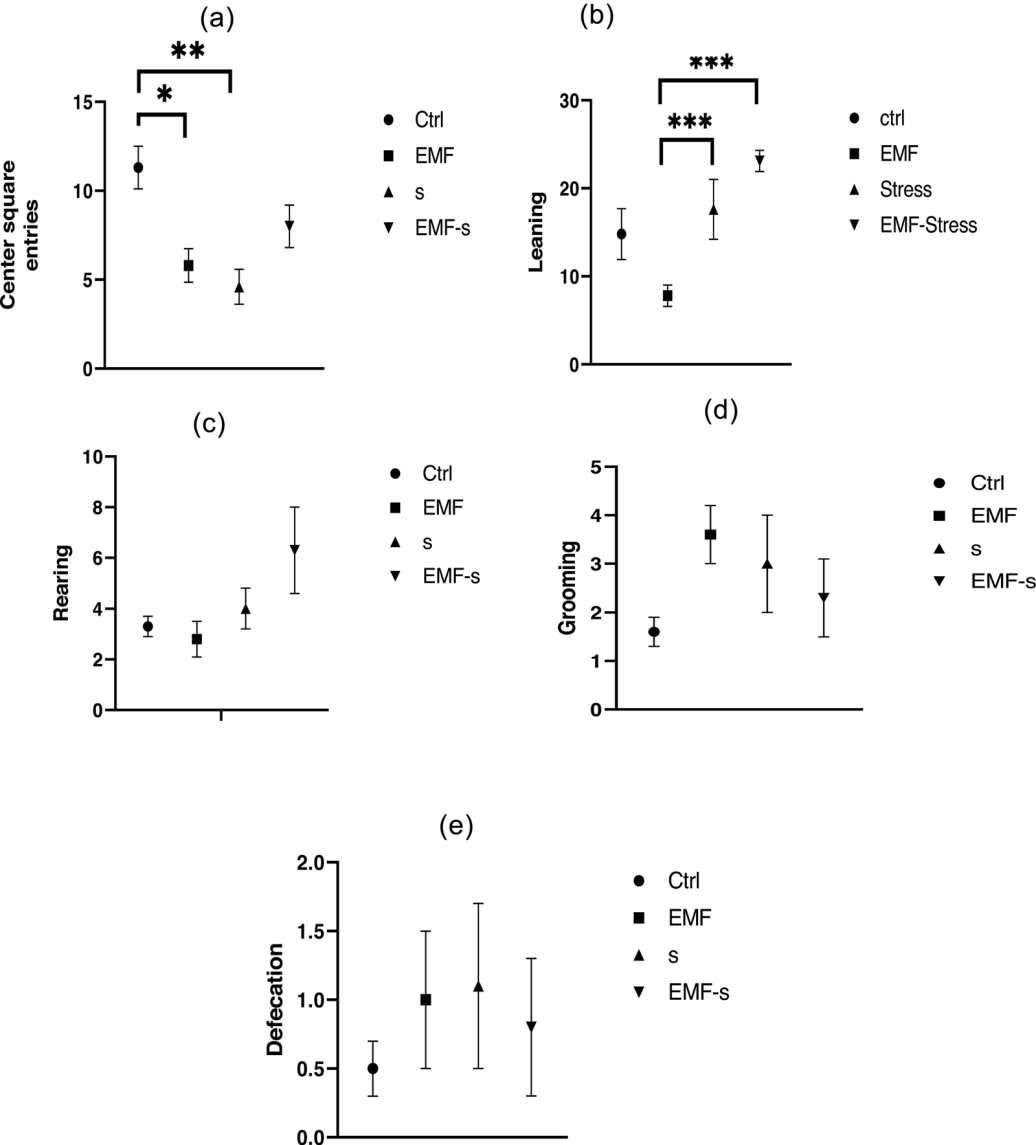
Effect of EMF on center square entries (a), leaning (b), rearing (c), grooming (d), defecation (e), after prenatal stress. Data are shown as mean ± SEM (*n* = 6 per group). Two‐way ANOVA, followed by Tukey's post hoc test: *p < .05, **p < .01, ***p < .001. ANOVA, analysis of variance; Ctrl, control; EMF, electromagnetic field; S, prenatal stress.

Center square entries reduced in EMF (5.8 ± 0.94) compared to control (11.3 ± 1.2) and also stress (4.6 ± 0.98) group showed significant decrease compared to control (*p* < 0.01). Leaning in stress (17.6 ± 3.4) and EMF/S (23.3 ± 1.2) groups was higher than EMF group (7.8 ± 1.2) (Figure 2).

### 3.3 The effect of ELF‐EMF and/or PS on expression of Capsa3, GAP‐43, and BDNF in prefrontal cortex (Figure 3)

**FIGURE 3:**
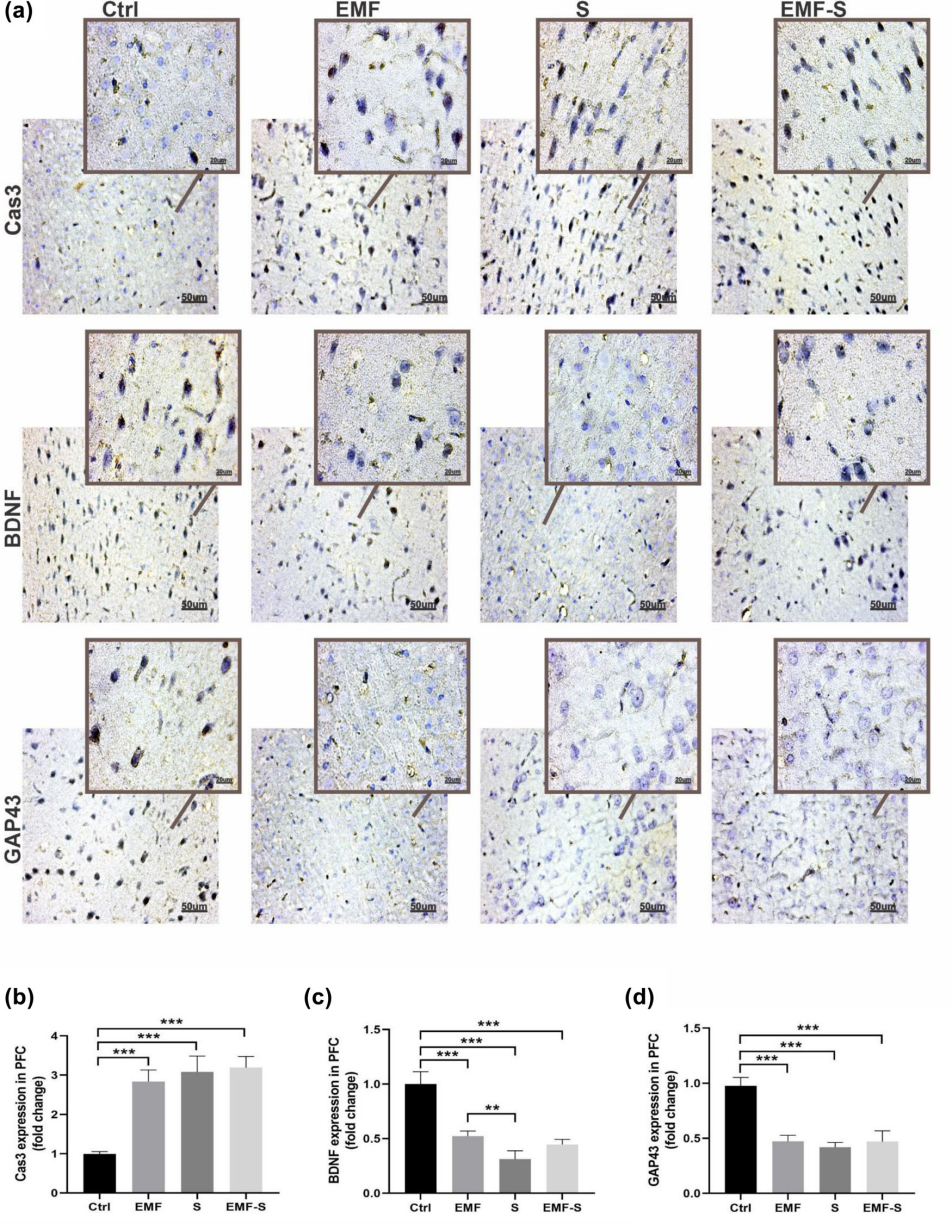
Immunohistochemistry analysis in the PFC. (a) Representative photomicrograph of immunohistochemistry. (b) Cas3‐positive area in PFC; (c) BDNF‐positive area in PFC; (d) GAP43‐positive area in PFC. All scale bars were 50 μm. The values represent the mean ± SEM,**p < 0.01, ***p < 0.001. Ctrl, control, EMF, extremely low‐frequency electromagnetic field, S, prenatal stress; EMF‐S: combined extremely low‐frequency electromagnetic field with prenatal stress; PFC, prefrontal cortex.

This protein was detected by immunohistochemisty method. Figure 2 shows that cas‐3 expression was upregulated in ELF‐EMF (2.83 ± 0.14), PS (3.08 ± 0.20), and ELF‐EMF/S (3.19 ± 0.14) compared to control (0.99 ± 0.02). BDNF expression was downregulated in ELF‐EMF (0.52 ± 0.02), PS (0.31 ± 0.03), and ELF‐EMF/S (0.44 ± 0.02) compared to control (1.00 ± 0.05). Similarly, GAP‐43 expression was downregulated in ELF‐EMF(0.47 ± 0.02), PS (0.41 ± 0.02), and ELF‐EMF/S (0.46 ± 0.04) compared to control (0.97 ± 0.037). Also, expression of BDNF in PS was considerably lower than ELF‐EMF (Figure 3).

### 3.4 The effect of ELF‐EMF and/or PS on expression of Capsa3, GAP‐43, and BDNF in DG (Figure 4)

**FIGURE 4:**
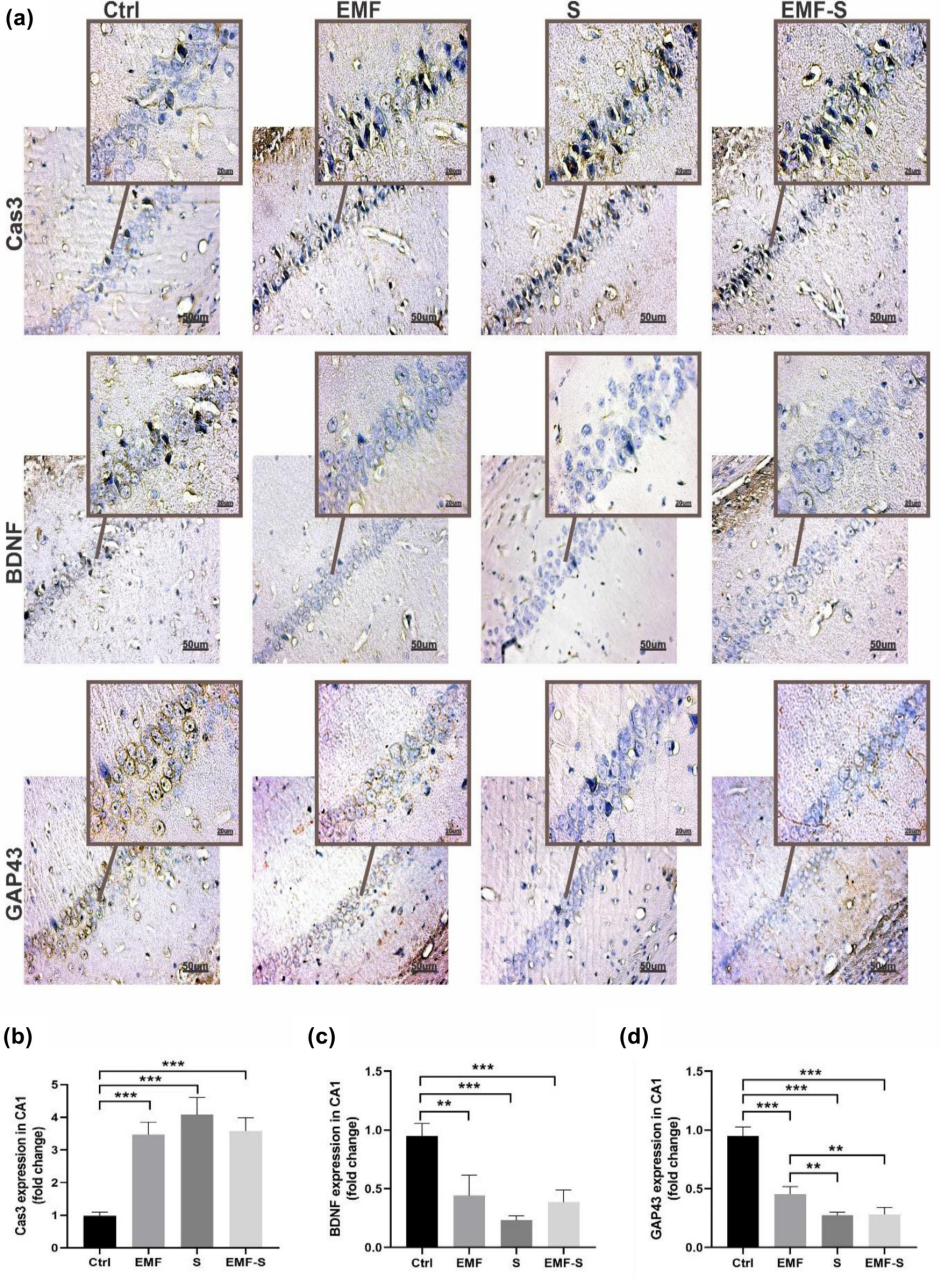
Immunohistochemistry analysis in the CA1 of hippocampus. (a) Representative photomicrograph of immunohistochemistry. (b) Cas3‐positive area, (c) BDNF‐positive area, (d) GAP43‐positive area. All scale bars were 50 μm. The values represent the mean ± SEM, **p < 0.01, ***p < 0.001. Ctrl, control; EMF, extremely low‐frequency electromagnetic field; S, prenatal stress; EMF‐S, combined extremely low‐frequency electromagnetic field with prenatal stress; PFC, prefrontal cortex; CA 1, Cornu Ammonis 1.

This protein was detected by immunohistochemisty method. Figure 3 shows that cas‐3 expression was upregulated in ELF‐EMF (1.82 ± 0.12), PS (2.39 ± 0.14), and ELF‐EMF/S (2.00 ± 0.12) compared to control (0.95 ± 0.05). BDNF expression was downregulated in ELF‐EMF (0.49 ± 0.02), PS (0.31 ± 0.01), and ELF‐EMF/S (0.37 ± 0.03) compared to control (0.95 ± 0.05). Similarly, GAP‐43 expression was downregulated in ELF‐EMF (0.36 ± 0.03), PS (0.35 ± 0.03), and ELF‐EMF/S (0.34 ± 0.01) compared to control (0.97 ± 0.05). Also, expression of cas‐3 in PS was significantly higher than ELF‐EMF and expression of BDNF in PS was considerably lower than ELF‐EMF (Figure 4).

### 3.5 The effect of ELF‐EMF and/or PS on expression of Capsa3, GAP‐43 and BDNF in CA1 (Figure 5)

**FIGURE 5:**
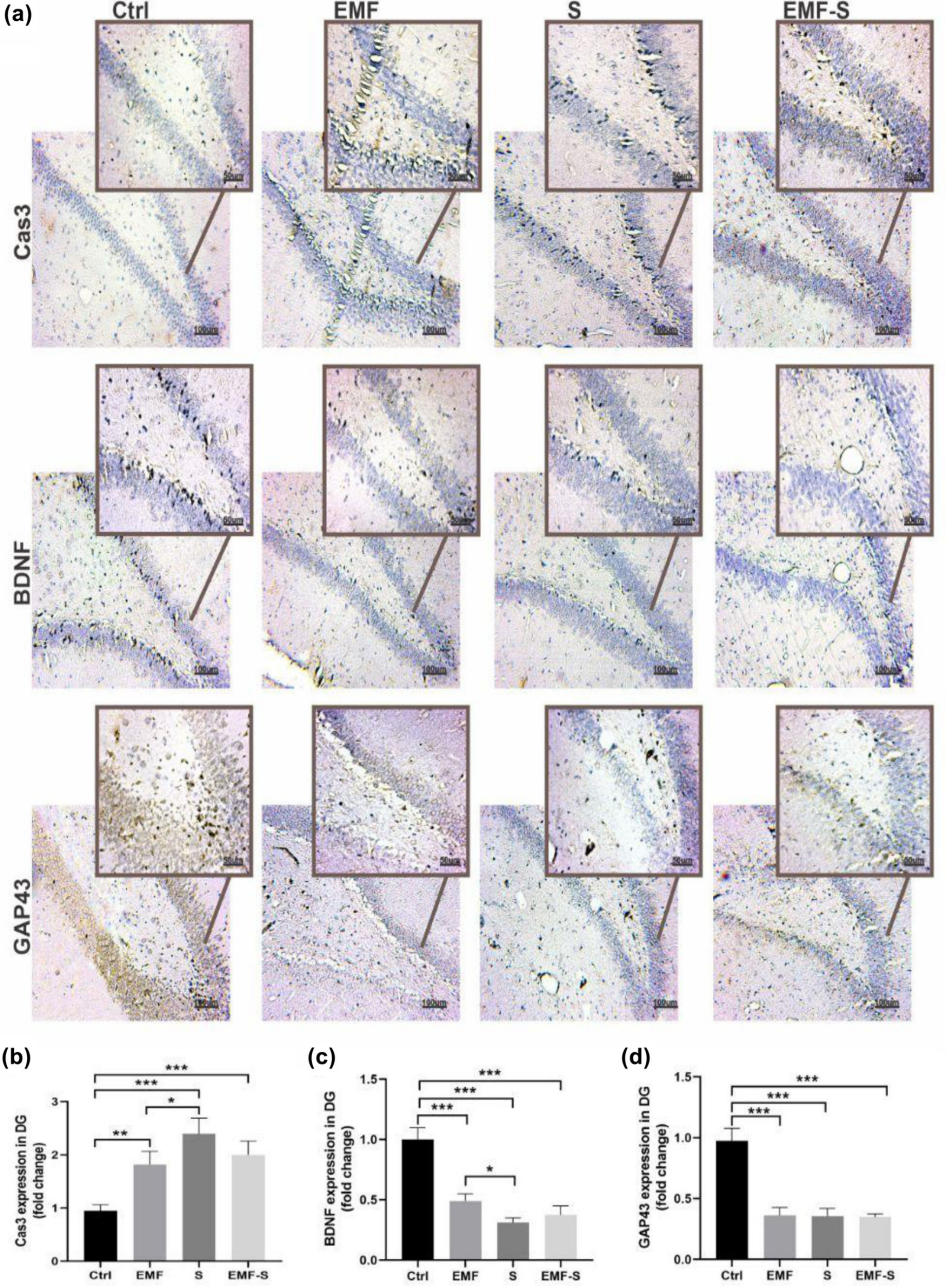
Immunohistochemistry analysis in the dentate gyrus of Hippocampus. (a) Representative photomicrograph of immunohistochemistry. (b) Cas3‐positive area in dentate gyrus, (c) BDNF‐positive area in dentate gyrus, (d) GAP43‐positive area in dentate gyrus. All scale bars were 50 μm. The values represent the mean ± SEM,*p < .05, **p < .01, ***p < .001. Ctrl, control; EMF, extremely low‐frequency electromagnetic field; S, prenatal stress; EMF‐S, combined extremely low‐frequency electromagnetic field with prenatal stress; DG, dentate gyrus.

This protein was detected by immunohistochemisty method. Figure 4 shows that cas‐3 expression was upregulated in ELF‐EMF (3.47 ± 0.19), PS (4.08 ± 0.26), and ELF‐EMF/S (3.58 ± 0.20) compared to control (0.98 ± 0.05). BDNF expression was downregulated in ELF‐EMF (0.44 ± 0.08), PS (0.23 ± 0.01), and ELF‐EMF/S (0.38 ± 0.05) compared to control (0.95 ± 0.05). Similarly, GAP‐43 expression was downregulated in ELF‐EMF(0.45 ± 0.03), PS (0.27 ± 0.01), and ELF‐EMF/S (0.28 ± 0.03) compared to control (0.95 ± 0.03). Also, expression of GAP43 in PS ELF‐EMF/S was considerably lower than ELF‐EMF (Figure 5).

### 3.6 The effect of PS and ELF‐EMF on histomorphomtry of PC (Figure 6)

**FIGURE 6:**
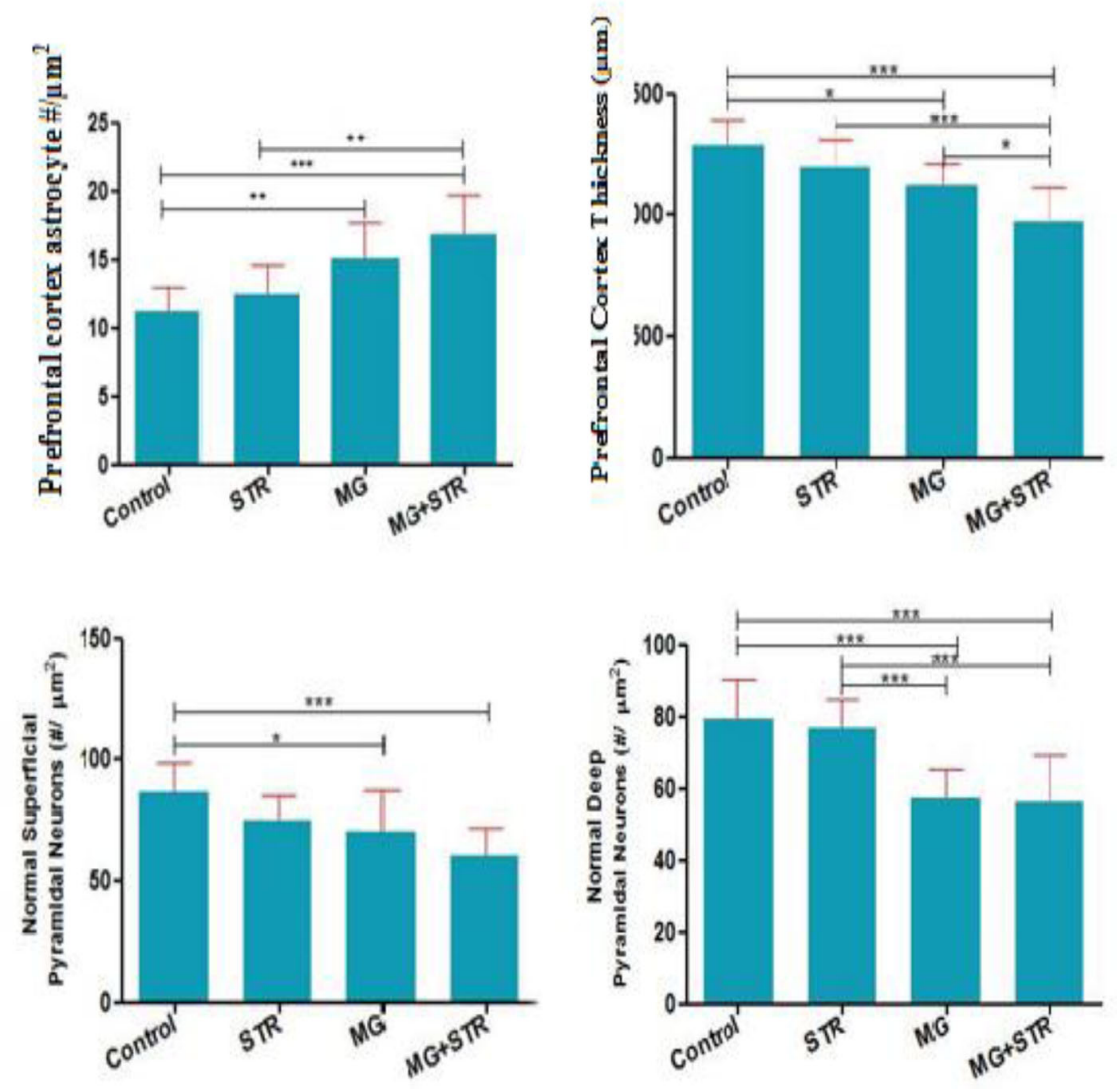
The effect of PS an ELF‐EMF on histomorphomtry of prefrontal cortex. Data are shown as mean ± SEM (*n* = 6 per group). Two‐way ANOVA, followed by Tukey's post hoc test: *p < .05, **p < .01, ***p < .001. ANOVA, analysis of variance; STR, stress; MG, extremly low‐frequency electromagnetic field.

Astrocytes number (#/μm^2^) increased in EMF (15.10 ± 2.64) and EMF/stress (16.80 ± 2.93) compared to control (11.20 ± 1.75). The number of superficial pyramidal neurons (#/μm^2^) in EMF (70.30 ± 17.06) and EMF/stress (60.50 ± 11.38) decreased compared to control (86.50 ± 12.13); also the number of deep pyramidal neurons (#/μm^2^) in EMF (57.10 ± 8.11) and EMF/stress (56.40 ± 12.86) decreased compared to control (79.20 ± 11.24). PC cortex thickness (μm) decreased in EMF (1122 ± 92.88) and EMF/stress (969.4 ± 144.8) compared to control (Figure 6).

### 3.7 The effect of PS and ELF‐EMF on histomorphomtry of DG, CA1, CA2, CA3, and CA4 (Figure 7)

**FIGURE 7:**
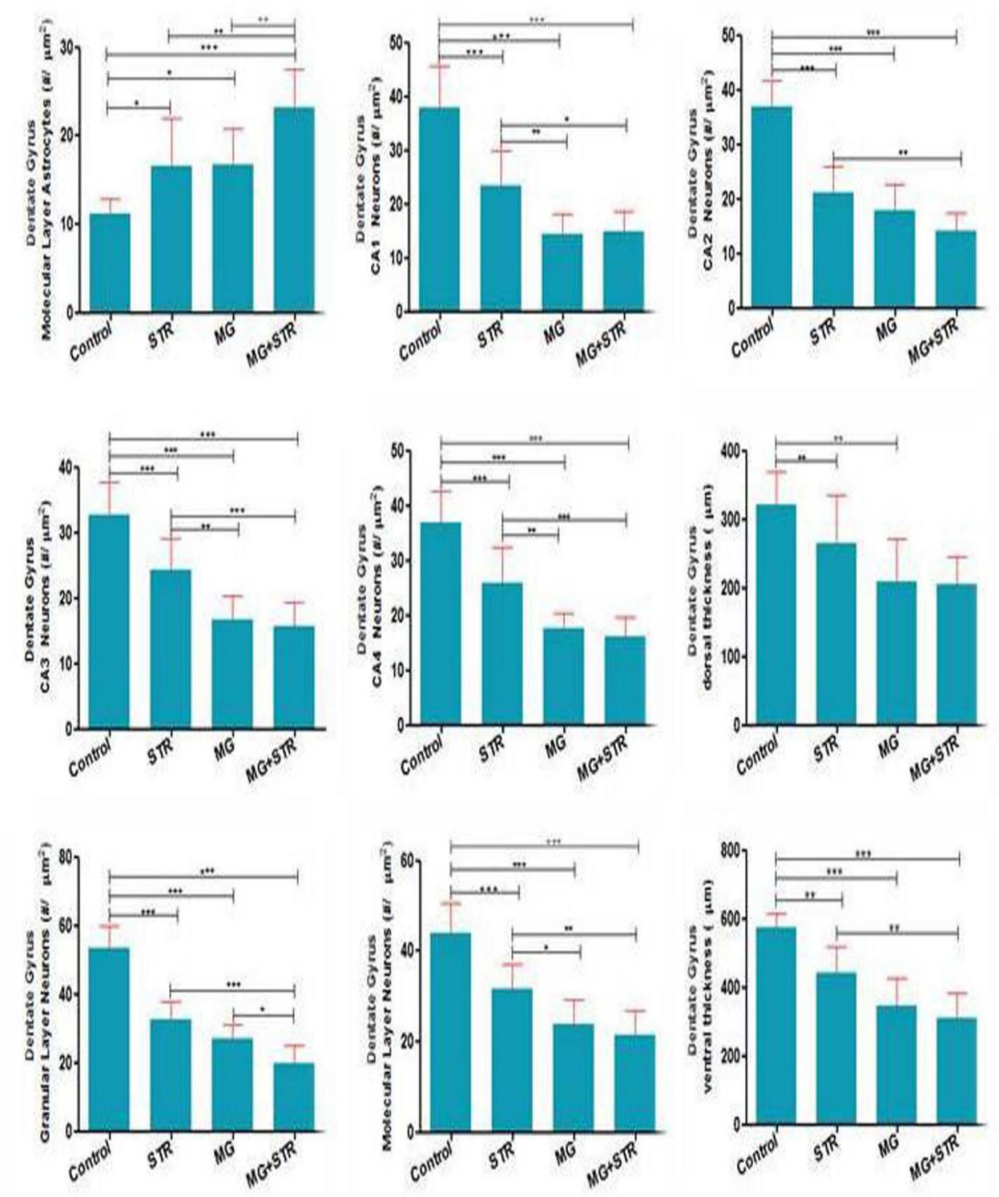
The effect of PS an ELF‐EMF on histomorphomtry of hippocampus. Data are shown as mean ± SEM (*n* = 6 per group). Two‐way ANOVA, followed by Tukey's post hoc test: *p < .05, **p < .01 ***p < 0.001. ANOVA, analysis of variance; STR, stress; MG, extremly low‐frequency electromagnetic field.

The number of dentate gyrus molecular layer astrocytes (#/μm^2^) increased in all treatment groups including EME (16.70 ± 4.11), stress (16.60 ± 5.44), and EMF/stress (23.20 ± 4.26) compared to control (11.10 ± 1.72). The number of neurons (#/μm^2^) in CA1 (EMF = 14.40 ± 3.71; stress = 23.40 ± 6.41; EMF/stress = 14.90 ± 3.69; control: 37.80 ± 7.88), CA2 (EMF = 21.00 ± 4.76; stress = 14.20 ± 3.29; EMF/stress = 17.80 ± 4.70; control: = 36.80 ± 1.54), CA3 (EMF = 16.60 ± 3.62; stress = 24.20 ± 4.91; EMF/stress = 15.70 ± 3.52; control = 32.70 ± 5.05), and CA4(EMF = 17.50 ± 2.75; stress = 25.80 ± 6.57; EMF/stress = 16.00 ± 3.55; control = 36.80 ± 5.77) decreased in all treatment groups compared to control. Also, dentate gyrus dorsal thickness (μm) decreased in EMF (209.1 ± 61.22) and stress (265.9 ± 68.73) compared to control (321.3 ± 48.40) and dentate gyrus ventral thickness (μm) decreased in all treatment including EME (347.1 ± 77.85), stress (442.1 ± 75.98), and EMF/stress (308.1 ± 75.30) compared to control (572.6 ± 42.86). The number of dentate gyrus granular layer neurons (#/μm^2^) decreased in all treatment groups including EMF (27.00 ± 3.88), stress (32.80 ± 4.89), EMF/stress (19.90 ± 5.10) compared to control (53.40 ± 6.39). The number of dentate gyrus molecular layer neurons (#/μm^2^) decreased in all treatment groups including EMF (31.70 ± 5.376), stress (23.90 ± 5.23), EMF/stress (21.40 ± 5.481) compared to control (43.90 ± 6.641) (Figure 7).

## 4 DISCUSSION

The main idea of our study was that ELF‐EMF as inducer of anxiety‐like behavior confirmed in many studies (Bagheri Hosseinabadi et al., 2019; Choleris et al., 2001; Djordjevic et al., 2017; Karimi et al., 2019; Liu et al., 2008) how would affect the well‐known anxiogenic effects of prenatal stress in offspring. We considered three modes for the outcome of this process. In this way, ELF‐EMF will not have any effect on the intensity of anxiety‐like behavior caused by prenatal stress, or it will aggravate this behavior, or it will interfere with the process of anxiogenic effect of prenatal stress and reduce anxiety‐like behavior. In the present study, we examined the effects of PS, ELF‐EMF, and simultaneously PS and ELF‐EMF on anxiety‐like behaviors, and expression of synaptic plasticity associated proteins (BDNF and GAP‐43) and neural cell apoptotic factor, cas‐3. As it was depicted previously (Hosseini et al., 2022), we observed that ELF‐EMF and stress groups showed anxiety‐like behavior. We found that female offspring which their mothers underwent PS and ELF‐EMF together, showed severe anxiety‐like behaviors, which were more intensive than those whose mother experienced only PS (group stress)  or only ELF‐EMF (group ELF‐EMF). Furthermore, anxiety‐like behaviors in all treatment groups were associated with increased expression cas‐3 and decreased expression of GAP3 and BDNF.  The present research revealed for the first time that PS combined with ELF‐EMF brought more serious anxiety‐like behaviors than only PS or only ELF‐EMF in female rats, and the neurodegeneration and synaptic plasticity deficiency of hippocampus and PFC may be the potential mechanism. This result is in accordance with a study that shows anxiety‐like behavior is correlated with reduction of neuroplasticity in the hippocampus and the PFC (Ieraci et al., 2016). We found a dramatic decrease in number of neurons in hippocampus and PFC in treatment groups, which can be considered as vast neurodegeneration. Neurodegeneration results in a decrease of neuroplasticity (Dorszewska et al., 2020) and subsequently anxiety‐like behavior (Ieraci et al., 2016) that we observed in present study. In addition, it has been found that synaptic signaling proteins regulate anxiety‐like behavior and have anxiolytic effect (Babaev et al., 2018) and hence, reduction of synaptic signaling proteins may have an anxiogenic effect. The decrease in synaptic signaling proteins has also been observed in anxiety‐like behavior (Diniz et al., 2021). Similarly, we observed a significant decrease in synaptic signaling proteins including GAP 43 and BDNF, which may be a reason why anxiety‐like behavior increased in treatment groups. There is conflicting evidence in that ELF‐EMF exposure has anxiogenic effects in rats. We observed an increase in anxiety‐like behavior in ELF‐EMF group. This is in consistent with some studies (Balind et al., 2016; Djordjevic et al., 2017; Shehu et al., 2016) and is in discrepancy with some others (Lai et al., 2016a; Rostami et al., 2016). This inconsistency can be due to the properties of the electromagnetic field such as power, frequency as well as the shape of the electromagnetic field, such as sinusoidal, squares, triangles, and saw teeth waves, as well as the duration of exposure can be another factor that can change the effect of the electromagnetic field. Our study showed that PS group in accordance with the majority of another studies (Kubo et al., 2018; Marrocco et al., 2012; Said et al., 2015; Vallée et al., 1997; Weinstock, 2017) exhibited an increase of anxiety‐like behavior compared to control.

Our results showed an increase of cas‐3 and reciprocal decrease of BDNF in DG and PFC in prenatally stressed offspring (Group Stress). Consistent with our study, chronic stress has been revealed to stimulate apoptosis of neurons through upregulation, increased cas‐3 and downregulation of brain‐derived neurotrophic factor (BDNF) (Dionisie et al., 2021). Moreover, chronic mild stressed mice showed increased cas‐3 in the hippocampus (Liu et al., 2010).

The hippocampus and PFC are key areas of the brain, which are influenced by stress (Carrier et al., 2021), and neurodegeneration is one of the implications of that. Additionally, PFC and hippocampus are the vital areas involved in anxiety‐like behavior (Bach et al., 2019; Jacobs & Moghaddam, 2021; Liu et al., 2020; Wang et al., 2019).

We observed vast neurodegeneration in DG, CA1, 2, 3, 4, and PFC in all treatment groups, accompanied by increased anxiety‐like behavior. In support, it should be noted that, on one hand, stress initiates neurodegeneration (Desmarais et al., 2020; Esch et al., 2002; Kline & Mega, 2020) and on the other hand, anxiety has been reported that is a common symptom in neurodegenerative diseases) Seignourel et al., 2008. (This shows that anxiety‐like behavior observed in prenatally stressed offspring (group stress) probably results from the vast neurodegeneration, which was occurred in our study. We also found that ELF‐EMF increased neurodegeneration and surprisingly exacerbated this process when it was accompanied with PS. Amazingly, neurodegeneration in all studied regions was coupled with upregulation of cas‐3 and downregulation of BDNF and GAP3. Neurodegeneration occurred in the hippocampus and PFC can be attributed to the over expression of cas‐3 and downregulation of BDNF and GAP3. From another point of view, decreased BDNF and GAP3 can reduce neuroplasticity in the hippocampus and the PFC, which may be another reason of anxiety‐like behavior observed in treatment groups.

Regarding the impact of ELF‐EMF, the results of our study are in conflict with studies that report the neurogenesis effects of ELF‐EMF (Cuccurazzu et al., 2010; Gao et al., 2021; Podda et al., 2014; Sakhaie et al., 2017b). To our knowledge, there is no study on the effects of prenatal ELF‐EMF on anxiety‐like behavior until now, and this is the first time that neurodegenerative effects of ELF‐EMF in the hippocampus and PFC are reported.

**FIGURE 8:**
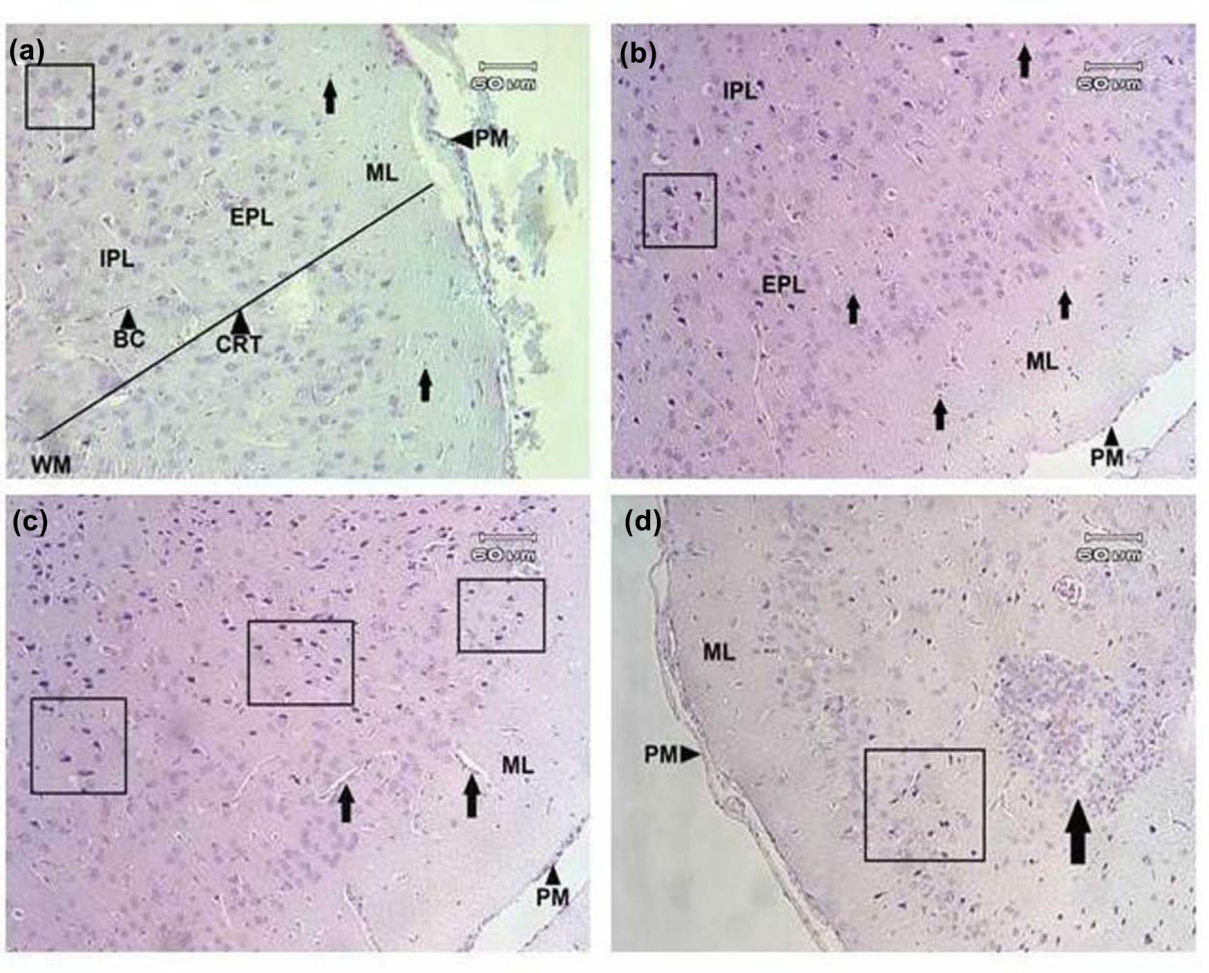
Coronal section of prefrontal cortex. (a) Control group. Regular cellular arrangement has been seen in different layers. Neurons with normal structure (inside square) with astrocytes (arrows) are observable. Pia matter (PM), molecular layer (ML), external pyramidal layer (EPL), internal pyramidal layer (IPL), CRT (cortical region), white matter (WM); blood capillary (BC). (b) Stress group. Structural alteration with nuclear condensation in pyramidal neurons is visible (inside square). Astrocytes (arrows). (c) ELF‐EMF group. Structural alterations are seen in neurons (inside squares). Increase of perivascular space (arrows) is visible. (d) ELF‐EMF/Stress group. Structural alterations are seen in neurons (inside square). H&E staining. Magnification 200×.

**FIGURE 9:**
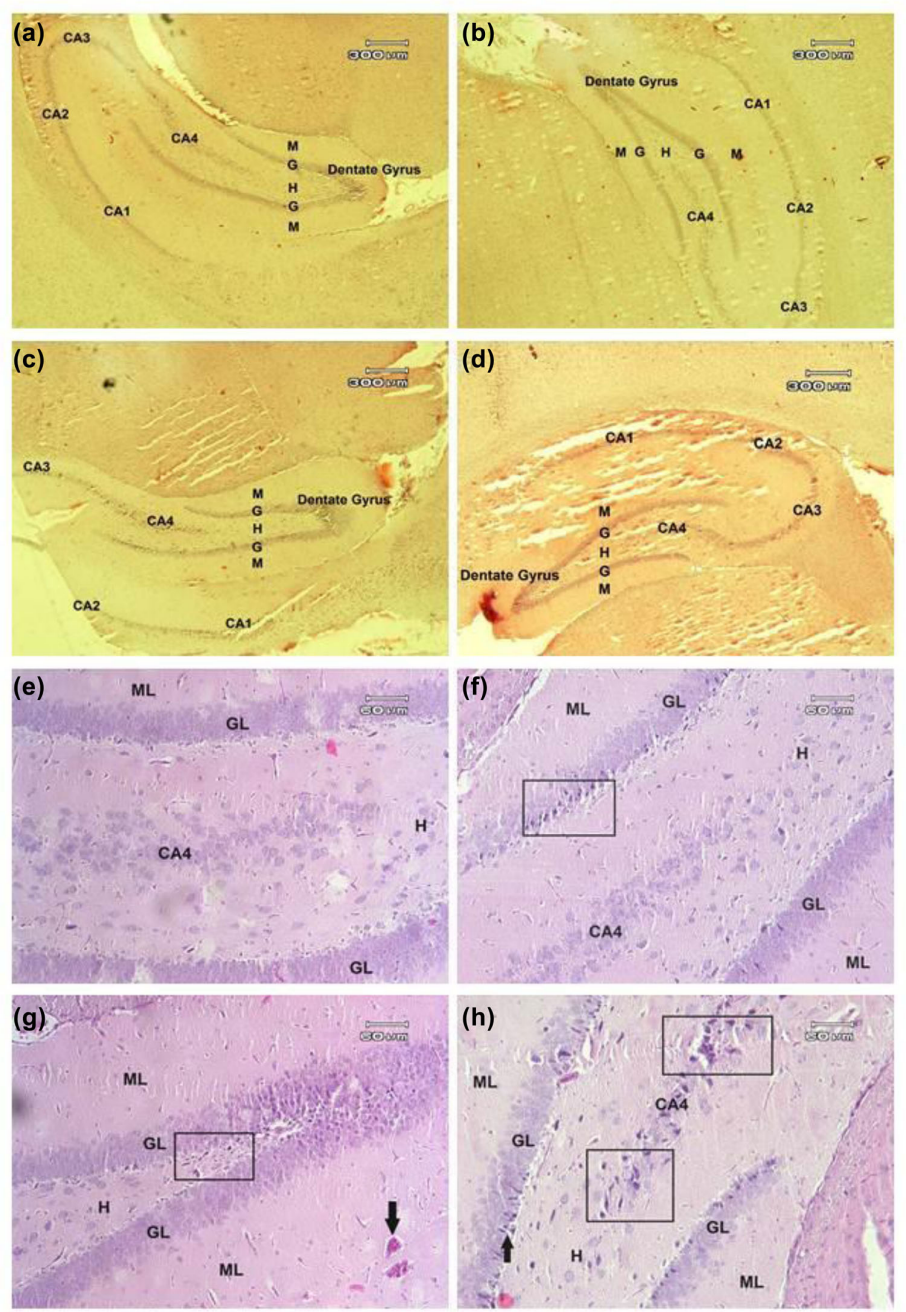
The structure of dentate gyrus in sagittal section of hippocampus. (a) Normal structure of neurons is visible in various parts. Hilus (H), molecular layer (M), granular layer (G), cornu ammonis (CA). (b, c, d) Stress, ELF‐EMF, and ELF‐EMF/stress groups respectively. (e) Higher magnification of dentate gyrus of control group with normal structure of various cellular layers. (f) Higher magnification of dentate gyrus of stress group. Structural alterations of neurons of granular layer is observable (inside square). Hilus (H), molecular layer (ML), granular layer (GL), the CA4 subfield of cornu ammonis (CA). (g) Higher magnification of dentate gyrus of ELF‐EMF field group. Alterations of neurons are observable (inside square). Vascular hyperemia (arrow) is visible. (h) Higher magnification of dentate gyrus of ELF‐EMF/Stress group. Structural alterations of neurons from CA4 are observable (inside squares). Nuclear condensation of granular neurons (arrow) is visible. H&E staining. Magnification a–d 40×, e–h 200×.

Moreover, we observed that astrocytes increased in DG and PFC, which can indicate an inflammatory state in these regions. We also detected structural alterations including nuclear condensation in neurons of CA4 in hippocamus, granular neurons of dentate gyrus and PFC that may result from increase of cas‐3 which initiate cell necrosis (Figures 8 and 9).

## 5 CONCLUSION

1. Our study showed that prenatal stress caused anxiety‐like behavior in offspring whose mothers underwent to stress. Also, ELF‐EMF induced anxiety‐like behavior in offspring whose mothers were exposed to ELF‐EMF and combination of prenatal stress and ELF‐EMF results in more severe anxiety‐like behavior in offspring whose mothers were simultaneously underwent to stress and exposed to ELF‐EMF.
2. The present study showed that dramatic increase in cas‐3 and decrease in BDNF, GAP‐ 43 in PFC and hippocampus caused by PS and ELF‐EMF could lead to significant neurodegeneration in hippocampus and PFC in the pregnancy period and could result in anxiety‐like behavior in offspring.
3. We also found for the first time that omnipresent ELF‐EMF not only induces neurodegeneration effects on hippocampus and PFC but also could exacerbate anxiety‐ like behavior of PS, which may be attributed to hippocampal and PFC neurodegeneration and also possible neuroplasticity reduction.

## AUTHOR CONTRIBUTIONS

Ehsan Hosseini designed study, performed treatments, and drafted and finalized manuscript. Davoud Kianifard performed molecular assays and histology studies and statistical analyses.

## FUNDING

There is not any financial support of special organization.

## CONFLICT OF INTEREST STATEMENT

The authors declare that they have not any conflict of interest.

### PEER REVIEW

The peer review history for this article is available at https://publons.com/publon/10.1002/brb3.2949.

## Data Availability

The data that support the findings of this study are available from the corresponding author upon reasonable request.
